# Patterns, predictors and prognostic relevance of high-grade hematotoxicity after temozolomide or temozolomide-lomustine in the CeTeG/NOA-09 trial

**DOI:** 10.1007/s11060-022-04203-4

**Published:** 2023-01-07

**Authors:** J. Weller, N. Schäfer, C. Schaub, T. Tzaridis, T. Zeyen, M. Schneider, A. L. Potthoff, F. A. Giordano, J. P. Steinbach, P. S. Zeiner, T. Kowalski, M. Sabel, P. Hau, D. Krex, O. Grauer, R. Goldbrunner, O. Schnell, G. Tabatabai, F. Ringel, F. Schmidt-Graf, S. Brehmer, J. C. Tonn, L. Bullinger, P. Vajkoczy, M. Glas, H. Vatter, U. Herrlinger, C. Seidel

**Affiliations:** 1 Division of Clinical Neurooncology, Department of Neurology and Center of Integrated Oncology (CIO), University Hospital Bonn, Venusberg-Campus 1, 53105 Bonn, Germany; 2 Department of Neurosurgery, University Hospital Bonn, Venusberg-Campus 1, Bonn, Germany; 3 Department of Radiation Oncology, University Medical Center Mannheim, Medical Faculty Mannheim, Heidelberg University, Mannheim, Germany; 4 Dr. Senckenberg Institute of Neurooncology, University of Frankfurt, Frankfurt, Germany; 5 Department of Neurology, University Hospital Knappschaftskrankenhaus, Ruhr–Universität Bochum, Bochum, Germany; 6 Department of Neurosurgery, University of Düsseldorf, Düsseldorf, Germany; 7 Department of Neurology and Wilhelm Sander NeuroOncology Unit, University Hospital Regensburg, Regensburg, Germany; 8 Department of Neurosurgery, University of Dresden, Dresden, Germany; 9 Department of Neurology, University of Münster, Münster, Germany; 10 Department of General Neurosurgery, Center of Neurosurgery, University of Cologne, Cologne, Germany; 11 Department of Neurosurgery, University of Freiburg, Freiburg, Germany; 12 Department of Neurology and Interdisciplinary Neurooncology, University Hospital Tübingen, Hertie Institute for Clinical Brain Research, Center for Neuro-Oncology, Comprehensive Cancer Center Tübingen-Stuttgart, German Cancer Consortium (DKTK), Partner site Tübingen, Eberhard Karls University, Tübingen, Germany; 13 Department of Neurosurgery, University of Mainz, Mainz, Germany; 14 Department of Neurology, Technical University of Munich, Munich, Germany; 15 Department of Neurosurgery, University of Mannheim, Mannheim, Germany; 16 Department of Neurosurgery, Ludwig Maximillian University of Munich and German Cancer Consortium (DKTK), Partner Site Munich, Munich, Germany; 17 Department of Hematology, Oncology and Tumorimmunology, Charité University of Berlin, Berlin, Germany; 18 Department of Neurosurgery, Charité University of Berlin, Berlin, Germany; 19 Division of Clinical Neurooncology, Department of Neurology and West German Cancer Center (WTZ), German Cancer Consortium, Partner Site Essen, University Hospital Essen, Essen, Germany; 20 Department of Radiation Oncology, University of Leipzig, Leipzig, Germany

**Keywords:** Glioblastoma, Temozolomide, Lomustine, MGMT, Hematotoxicity

## Abstract

**Purpose:**

In the randomized phase III trial CeTeG/NOA-09, temozolomide (TMZ)/lomustine (CCNU) combination therapy was superior to TMZ in newly diagnosed MGMT methylated glioblastoma, albeit reporting more frequent hematotoxicity. Here, we analyze high grade hematotoxicity and its prognostic relevance in the trial population.

**Methods:**

Descriptive and comparative analysis of hematotoxicity adverse events ≥ grade 3 (HAE) according to the Common Terminology of Clinical Adverse Events, version 4.0 was performed. The association of HAE with survival was assessed in a landmark analysis. Logistic regression analysis was performed to predict HAE during the concomitant phase of chemotherapy.

**Results:**

HAE occurred in 36.4% and 28.6% of patients under CCNU/TMZ and TMZ treatment, respectively. The median onset of the first HAE was during concomitant chemotherapy (i.e. first CCNU/TMZ course or daily TMZ therapy), and 42.9% of patients with HAE receiving further courses experienced repeat HAE. Median HAE duration was similar between treatment arms (CCNU/TMZ 11.5; TMZ 13 days). Chemotherapy was more often discontinued due to HAE in CCNU/TMZ than in TMZ (19.7 vs. 6.3%, p = 0.036). The occurrence of HAE was not associated with survival differences (p = 0.76). Regression analysis confirmed older age (OR 1.08) and female sex (OR 2.47), but not treatment arm, as predictors of HAE.

**Conclusion:**

Older age and female sex are associated with higher incidence of HAE. Although occurrence of HAE was not associated with shorter survival, reliable prediction of patients at risk might be beneficial to allow optimal management of therapy and allocation of supportive measures.

**Trial registration:**

NCT01149109.

## Introduction

Glioblastoma is the most common malignant primary brain tumor in adults and has a detrimental prognosis despite standard-of-care treatment with surgery, radiotherapy, and temozolomide chemotherapy [1]. The presence of *O*^*6*^*-methylguanine-DNA methyltransferase* (*MGMT*) promotor methylation defines a subgroup with prolonged survival and benefit from temozolomide (TMZ) [2]. CeTeG/NOA-09, a randomized phase III trial in patients with glioblastoma harboring a methylated *MGMT* promotor investigated a combination chemotherapy with TMZ and lomustine (CCNU) in addition to standard-of-care surgery and radiotherapy and was able to show an increase in median overall survival from 31.4 months to 48.1 months [3]. Within this trial, hematologic toxicity was more frequent in the combined treatment arm and fewer patients were able to complete all courses of chemotherapy, potentially leading to concerns regarding safety upon implementation of this therapy, while health-related quality of life was unaffected [4].

The aim of the present study is to provide a detailed analysis of patterns, predictors and prognostic effect of high grade hematologic adverse event (HAE) in the CeTeG/NOA-09 trial.

## Methods

### Study design, participants and treatment

The CeTeG/NOA-09 study design has been published previously [3]. Briefly, this German multicenter, randomized, open-label, phase III trial enrolled patients aged 18–70 years with newly diagnosed, histologically confirmed, chemotherapy-naïve, *MGMT* promotor methylated glioblastoma and a Karnofsky performance score of 70 or higher. Adequate hematological, hepatic, renal, and coagulation function and absence of medical treatment for any cancer were among inclusion criteria [3]. All patients provided written informed consent and the study was approved by the ethics committees of all participating centers. Patients were planned to receive standard focal radiotherapy (total 60 Gy) in addition to either standard oral TMZ (concomitant daily 75 mg/m^2^, followed by six courses of 150–200 mg/m^2^ for 5 days every 4 weeks) [5], or six 6-week courses of oral combined CCNU/TMZ (CCNU 100 mg/m^2^ on day 1, TMZ 100–200 mg/m^2^ on days 2–6) starting in the first week of radiotherapy. As described previously, dose modifications of CCNU/TMZ were performed according to results of mandatory weekly blood tests. If the nadir (white blood count < 1500 cells/µl or platelets < 50,000/µl) occurred after day 25, CCNU was reduced stepwise (steps in mg/m^2^: 100, 75, 50, 0). TMZ dose was adapted according to the nadir during the first 25 days of the preceding course. Starting from 100 mg/m^2^ in the first course, TMZ dose was increased to 120, 150, 200 mg/m^2^ (maximum dose) in the next courses if no relevant hematotoxicity was observed. If the TMZ-related white blood count nadir was < 1500 cells/µl or platelet count < 50,000/µl, the TMZ dose of the next course was decreased by one step of the possible dose levels 200, 150, 120, 100, 75, 50, 0 mg/m^2^ or decreased by two steps if white blood count was < 1000/µl or platelet count < 25,000/µl. If a course was delayed for more than 6 weeks, study therapy was discontinued. Non-hematological toxicity grade 3 or 4 led to discontinuation of the causing substance. All patients were followed up with clinical examination and MRI every 3 months.

HAE were defined as thrombopenia, leukopenia, lymphopenia, neutropenia, or anemia of grade 3 or higher. All adverse events were rated according to the Common Terminology of Clinical Adverse Events (CTCAE) version 4.0 and documented using pre-specified clinical reporting forms.

### Statistical analysis

Standard descriptive statistics were used for all presented data. Group differences were analyzed with Fisher’s exact test for categorical variables, and Mann-Whitney U test for continuous and ordinal variables as normal distribution could not be assumed.

Survival analysis was performed using Cox regression to analyze the impact of HAE and a delay of chemotherapy courses by 2–6 weeks. Given the time-dependent nature of HAE and delayed chemotherapy courses, i.e. an increasing cumulative incidence in patients receiving longer treatment, a landmark analysis was performed [6]. Three landmark times were specified: the end of the concomitant phase, 3rd and 6th chemotherapy course for the analysis of HAE, and the initiation of the 2nd, 4th and 6th course for the analysis of delay. Patients receiving chemotherapy until the respective landmark time were included, and the landmark datasets were stacked. Cox models were stratified by landmark times.

Uni- and multivariable logistic regression was performed to predict HAE during the concomitant phase of chemotherapy using previously published parameters [7, 8]. Significance level was set to alpha = 0.05 and all analyses were two-sided. Statistical calculations were carried out with SPSS (version 25, IBM Corp., Armonk, NY) and R (version 4.2, R core team 2022).

## Results

### Patterns of hematologic adverse events grade 3 or higher

**Fig. 1:**
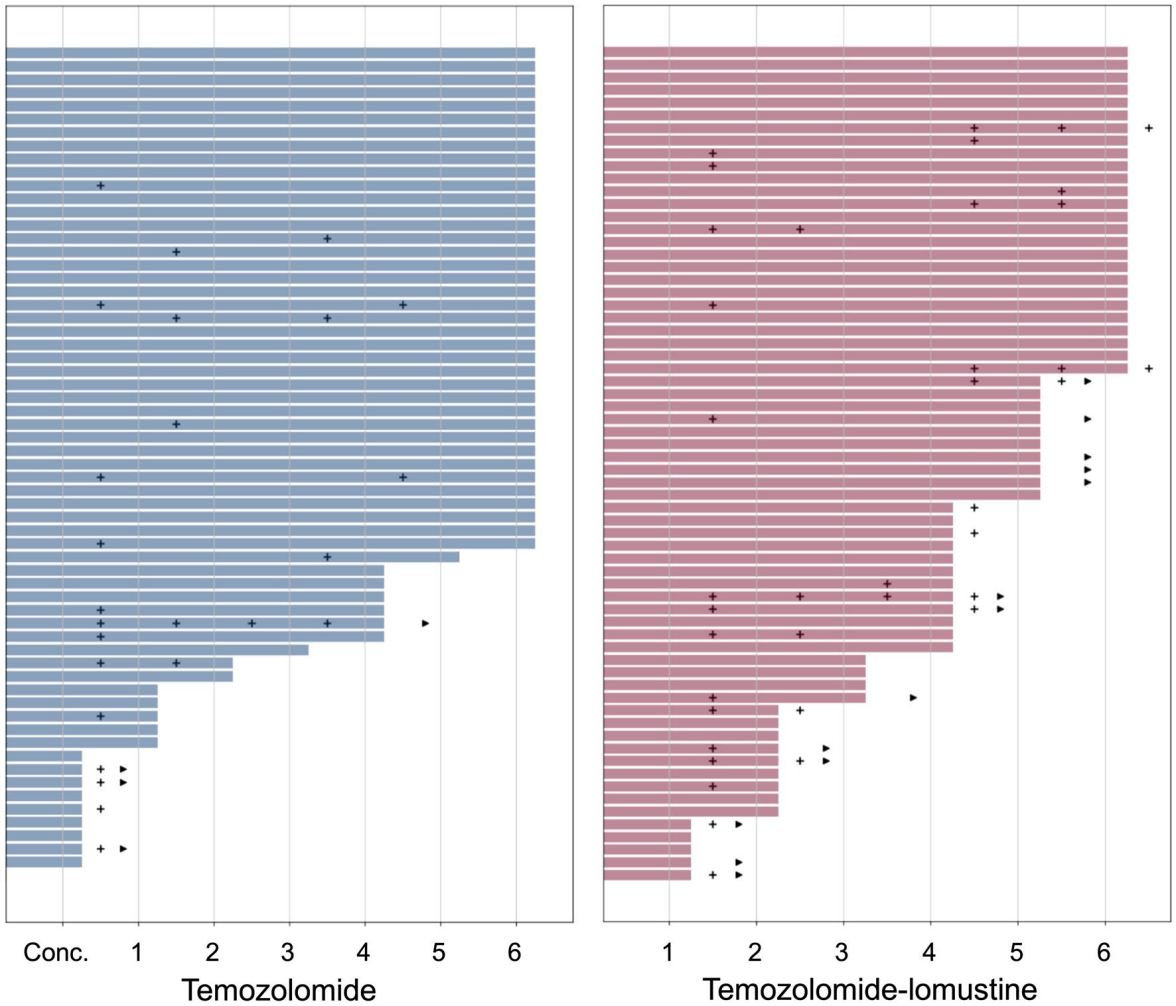
Swimmer plot with individual patient data on applied chemotherapy courses and hematotoxicity. Crosses indicate hematological adverse events CTCAE grade 3 or 4, triangles indicate discontinuation of chemotherapy due to hematotoxicity or a resulting delay of more than 6 weeks. *conc*, *concomitant temozolomide*

The prevalence of HAE was higher in patients treated with CCNU/TMZ compared to treatment with TMZ alone without reaching statistical significance (36.4% (24 of 66) vs. 28.6% (18 of 63), respectively, p = 0.36) as reported before [3]. The median onset of a patient’s first HAE was the concomitant phase of chemotherapy for both treatment arms (CCNU/TMZ: 1st course, interquartile range [IQR] 1–4, TMZ: conc. course, IQR: conc.–6th course, Fig. 1). In the concomitant phase of chemotherapy, HAEs occurred in 22.7% (15 of 66) of patients treated with CCNU/TMZ and 20.6% (13 of 63) patients treated with TMZ (p = 0.83).

The duration of HAE in the concomitant phase of chemotherapy was longer in the TMZ arm (concomitant: median 20 days, IQR 13–36.5, n = 13) compared to the CCNU/TMZ arm (1st course: median 10 days, IQR 2–24, n = 15) without reaching statistical significance (p = 0.10). We observed the opposite for HAE during adjuvant courses; the median duration in TMZ was 6.5 days (IQR 4.5–13.25), compared to CCNU/TMZ with 20 days (IQR 11.25–51.5, p = 0.004). For all HAE episodes combined, the median duration was 11.5 days (IQR 5.25–22.75) in the CCNU/TMZ arm and 13 days (IQR 6.75–23.25) in the TMZ arm, respectively (p = 0.46). The duration of lymphopenia grade 3 or 4 was longer under CCNU/TMZ treatment, but this observation was based on a low number of cases (Table 1). No significant differences in duration were found for other HAEs (Table 1).

**Table 1:** Median duration and interquartile range (IQR) of hematotoxicity grade 3 or 4 episodes

	Lomustine-Temozolomide		Temozolomide		
	n (%)	Median duration (IQR)	n (%)	Median duration (IQR)	p
Leukopenia	10 (15.2)	9 (6 – 16)	8 (12.7)	9 (6–21)	0.76
Neutropenia	8 (12.1)	10.5 (8 – 14.75)	4 (6.3)	11 (5–21.5)	0.95
Thrombopenia	19 (28.8)	13 (4.25–20.5)	15 (23.8)	14.5 (5.5–31.75)	0.46
Lymphopenia	3 (4.5)	140 (90 – n.d.)	4 (6.3)	7 (6.5–21.5)	0.024
Anemia	1 (1.5)	2 (n.d. – n.d.)	3 (4.8)	12.5 (1 – n.d.)	0.99

High risk neutropenia, defined as CTCAE grade 4 neutropenia > 7 days [9], occurred in 1 patient in the CCNU/TMZ arm, and no case of febrile neutropenia was recorded. No hematologic adverse event grade 5 occurred.

### Risk of recurring hematotoxicity

Among patients experiencing HAE, the median number of events was 1 (range, 1–4), with no difference between treatment arms (TMZ: 1 [range 1–4], CCNU/TMZ: 1 [range 1–4], p = 0.40). The risk of repeat HAEs during later courses among patients receiving at least one further course of chemotherapy after first HAE was 42.9% (15 of 35), numerically larger with CCNU/TMZ (47.6%, 10 of 21) compared to TMZ (35.7%, 5 of 14, p = 0.73).

### Impact of hematotoxicity on chemotherapy and survival

Chemotherapy was completed (i.e. concomitant + 6 adjuvant courses of TMZ or 6 courses of CCNU/TMZ) in 40.6% (26 of 66) of patients receiving CCNU/TMZ and 59.4% (38 of 63) of patients receiving TMZ, as reported before [3]. Among patients receiving at least two courses of CCNU/TMZ or concomitant + first adjuvant course of TMZ, dose reductions were performed in 31.6% (36 of 114 patients); 36.1% (22 of 61) with CCNU/TMZ and 26.4% (14 of 53) with TMZ (p = 0.32).

According to protocol, chemotherapy was stopped if a course was delayed by more than 6 weeks. More patients stopped chemotherapy due to hematotoxicity or resulting delay under CCNU/TMZ (19.7%, 13 of 66) than TMZ (6.3%, 4 of 63, p = 0.0358, Fig. 1). The preceding hematotoxicity was grade 3 or 4 in all cases for TMZ but only in 53.8% of cases (7 of 13) for CCNU/TMZ, in the remaining cases lower grade hematotoxicity caused a delay of > 6 weeks. Chemotherapy was mostly stopped directly after the concomitant course of TMZ (75%, 3 of 4 patients) compared to later courses in CCNU/TMZ (first course: 20%, 3 of 15 patients).

The occurrence of HAE was not associated with shorter survival in the entire cohort (hazard ratio [HR] 1.06, 95% CI 0.73–1.54, p = 0.76) nor for both arms separately (TMZ: HR 1.08, 95% CI 0.63–1.85, p = 0.79, CCNU/TMZ: HR 1.03, 95% CI 0.61–1.75, p = 0.91).

Survival was also similar in patients with a delay of 2–6 weeks for any course of chemotherapy compared to patients without such a delay, both in the entire cohort (HR 0.70, 95% CI 0.43–1.16, p = 0.17) and for both arms separately (TMZ: HR 0.59, 95% CI 0.29–1.21, p = 0.15; CCNU/TMZ: HR 0.82, 95% CI 0.40–1.66, p = 0.58).

### Prediction of hematotoxicity

In univariable logistic regression analysis, older age and female sex were associated with HAE (Table 2). Other previously published baseline parameters[7, 8], including reduced platelet counts, steroid or bowel medication and elevated creatinine had no predictive value for the occurrence of HAE. Analyzing either arms separately, no significant predictors were identified (Table 2). Multivariable analysis confirmed both female sex (odds ratio: 2.63, 95% CI 1.03–7.71, p = 0.043) and older age (odds ratio per year increment: 1.08, 95% CI 1.02–1.15, p = 0.01) as significant predictors for HAE.

**Table 2:** Univariable logistic regression analysis identifies female sex and older age as predictors for hematotoxicity. Abbreviations: OR, odds ratio; CI, confidence interval; PPI, proton pump inhibitior treatment

	Pooled			Lomustine-Temozolomide			Temozolomide		
	OR	95% CI	P	OR	95% CI	p	OR	95% CI	p
Female sex	2.47	1.00–6.10	0.050	3.15	0.87–11.48	0.08	2.08	0.56–7.79	0.28
Age	1.08	1.02–1.15	0.011	1.09	0.99–1.19	0.06	1.08	0.99–1.18	0.09
Creatinine > 1 mg/dl	0.21	0.02–1.64	0.14	0.35	0.04–2.99	0.35	0.00	0–0	0.99
Platelets < 270/µl	0.37	0.27–1.62	0.37	0.77	0.22–2.69	0.68	0.56	0.15–2.11	0.39
PPI	0.69	0.24–2.02	0.50	0.67	0.14–3.64	0.67	0.58	0.16–2.79	0.67
Steroid treatment at baseline	1.01	0.26–3.87	0.99	2.5	0.40–15.56	0.33	0.42	0.48–3.72	0.44
Experimental arm	0.94	0.39–2.29	0.90	–	–	–	–	–	–

*OR* odds ratio, *CI* confidence interval, *PPI* proton pump inhibitior treatment

## Discussion

The present study provides a detailed comparative analysis of high grade hematotoxicity in the CeTeG/NOA-09 trial. No hematologic adverse event grade 5 was reported and the overall frequency of HAE showed a non-significant tendency to be higher with CCNU/TMZ as compared to TMZ [3]. Interestingly, the risk of HAE was highest during the first phase of chemotherapy (first course of CCNU/TMZ or concomitant TMZ, both during radiotherapy) with a similar frequency for both treatment arms, and the same number of patients in both arms discontinued therapy due to hematotoxicity as a consequence of the first phase of chemotherapy. The risk for repeat HAE during later courses was high (42.9%) despite dose adjustment required by protocol, and affected patients should be monitored closely.

HAE patterns differed between the treatment arms: compared to TMZ, more patients developed HAE during later courses in the CCNU/TMZ arm. HAE in the TMZ arm lasted longest after concurrent treatment, while HAE from CCNU/TMZ were more prolonged during later courses. Most likely, the 6-week long exposure to TMZ during the concomitant phase of radiochemotherapy (employing > 1/3 of the total maximum temozolomide dose given during therapy) translates to a longer lasting nadir than the adjuvant 5/28 courses. On the other hand, CCNU/TMZ courses with increasing intensity of the CCNU/TMZ treatment scheme and potentially cumulative and prolonged toxicity of CCNU may explain the more frequent HAE onset in later courses. Consequently, more patients in the CCNU/TMZ arm compared to TMZ discontinued chemotherapy due to hematotoxicity or a resulting delay of > 6 weeks.

In line with previous reports, the occurrence of HAE was not associated with shorter survival in our analysis [10]. Nevertheless, the question is raised if secondary prophylactic measures enabling application of further courses in patients at risk of discontinuing therapy, e.g. romiplostim treatment after severe thrombopenia [11], might improve outcome.

The duration of HAE was similar between treatment arms with the exception of lymphopenia, which lasted longer in CCNU/TMZ than in TMZ. However, this observation was based on a small number of observations and lymphopenia is often perceived as a less threatening HAE due to the possibility of chemoprophylaxis for pneumocystis jirovecii pneumonia [12]. The overall low incidence of lymphopenia grade 3 or 4 in both treatment arms raises the possibility of underreporting of this specific HAE, as much higher frequencies of lymphopenia grade 3 or 4 have been reported in TMZ-treated glioblastoma patients [13].

Comparing HAE rates to former studies, one has to consider the different follow up schemes for blood tests. The present study required mandatory weekly blood tests to attribute hematotoxic events to the suspected causative chemotherapeutic drug and guide dose adjustment. This careful follow-up could explain the higher rate of HAE during standard TMZ treatment of 29%, compared to 16% upon monthly examination in the landmark EORTC 22981/26981-NCIC CE3 trial [5].

We also evaluated the prediction of hematotoxicity employing previously published baseline factors found to be predictive in glioma patients receiving TMZ chemotherapy [7, 8]. Logistic regression analysis confirmed the known higher risk among older and female patients [14], while treatment arm was not predictive. Analyzing treatment arms separately, no significant predictors were found, probably due to limited sample size and event rate. Although the analysis of health-related quality of life data showed no detrimental effect of CCNU/TMZ in the trial, adverse events should be minimized as much as possible, without reducing clinical efficacy [4].

The association of female gender and increased risk of HAE has been reported for temozolomide in glioma treatment [7, 14, 15], but also for other chemotherapeutics agents employed in a variety of cancers [16]. Indeed, sex differences in pharmacologic response and adverse drug reactions are increasingly observed and female sex is associated with a greater risk of adverse events [17]. These observations are attributed to differences in pharmacokinetics and pharmacodynamics [18]. Females have a higher percentage of body fat, affecting distribution volumes, but obesity and body fat content were not correlated with myelosuppression [14]. Other potential mechanisms underlying the sex-dependently increased HAE risk include a lower glomerular filtration rate and activity of hepatic enzymes and drug transporters, which may affect drug clearance [16, 17]. While these aspects demand further investigation, recommended dose adjustments and monitoring for HAE in female and elderly patients carrying the highest risk for HAE seems paramount [3].

In addition to clinical factors, little is known about the contribution of genetic factors to hematotoxicity susceptibility. Lombardi et al. found a methylated *MGMT* promoter in blood cells of patients with severe hematologic toxicity [8], and single nucleotide polymorphisms of *MGMT* are under observation [19]. Prospective evaluation of these factors in larger series is needed to better understand the molecular basis of hematotoxicity.

## Conclusion

This detailed analysis of HAE in the CeTeG/NOA-09 trial supports the clinical follow-up and management of patients treated with CCNU/TMZ or TMZ. We conclude that close monitoring is mandatory, and potentially supportive measures might be helpful. Importantly, HAE was not associated with shorter survival. Increased HAE risk in older and female patients was confirmed, but reliable prediction of HAE should be further examined in larger studies.

## Data Availability

The dataset is available from the corresponding author upon reasonable request. The data are not publicly available due to privacy restrictions.

## References

[1] Wen PY, Weller M, Lee EQ (2020). Glioblastoma in adults: a Society for Neuro-Oncology (SNO) and european society of neuro-oncology (EANO) consensus review on current management and future directions. Neuro Oncol.

[2] Hegi ME, Diserens A-C, Gorlia T (2005). MGMT gene silencing and benefit from temozolomide in glioblastoma. N Engl J Med.

[3] Herrlinger U, Tzaridis T, Mack F (2019). Lomustine-temozolomide combination therapy versus standard temozolomide therapy in patients with newly diagnosed glioblastoma with methylated MGMT promoter (CeTeG/NOA-09): a randomised, open-label, phase 3 trial. Lancet.

[4] Weller J, Tzaridis T, Mack F (2019). Health-related quality of life and neurocognitive functioning with lomustine-temozolomide versus temozolomide in patients with newly diagnosed, MGMT-methylated glioblastoma (CeTeG/NOA-09): a randomised, multicentre, open-label, phase 3 trial. Lancet Oncol.

[5] Stupp R, Mason WP, van den Bent MJ (2005). Radiotherapy plus concomitant and adjuvant temozolomide for glioblastoma. N Engl J Med.

[6] Morgan CJ (2019). Landmark analysis: a primer. J Nucl Cardiol.

[7] Armstrong TS, Cao Y, Scheurer ME (2009). Risk analysis of severe myelotoxicity with temozolomide: the effects of clinical and genetic factors. Neurooncology.

[8] Lombardi G, Rumiato E, Bertorelle R (2015). Clinical and genetic factors associated with severe hematological toxicity in glioblastoma patients during radiation plus temozolomide treatment: a prospective study. Am J Clin Oncol.

[9] Freifeld AG, Bow EJ, Sepkowitz KA (2011). Clinical practice guideline for the use of antimicrobial agents in neutropenic patients with cancer: 2010 update by the infectious diseases society of America. Clin Infect Dis.

[10] Le Rhun E, Oppong FB, Vanlancker M (2022). Prognostic significance of therapy-induced myelosuppression in newly diagnosed glioblastoma. Neuro-Oncol.

[11] Le Rhun E, Devos P, Houillier C (2019). Romiplostim for temozolomide-induced thrombocytopenia in glioblastoma: the PLATUM trial. Neurology.

[12] Cooley L, Dendle C, Wolf J (2014). Consensus guidelines for diagnosis, prophylaxis and management of *P neumocystis jirovecii* pneumonia in patients with haematological and solid malignancies, 2014: guidelines for prevention and treatment of PJP. Intern Med J.

[13] Stupp R, Dietrich P-Y, Ostermann Kraljevic S (2002). Promising survival for patients with newly diagnosed glioblastoma multiforme treated with concomitant radiation plus temozolomide followed by adjuvant temozolomide. J Clin Oncol.

[14] Zeiner PS, Filipski K, Filmann N (2022). Sex-dependent analysis of temozolomide-induced myelosuppression and effects on survival in a large real-life cohort of patients with glioma. Neurology.

[15] Gupta T, Mohanty S, Moiyadi A, Jalali R (2013). Factors predicting temozolomide induced clinically significant acute hematologic toxicity in patients with high-grade gliomas: a clinical audit. Clin Neurol Neurosurg.

[16] Özdemir BC, Csajka C, Dotto G-P, Wagner AD (2018). Sex differences in efficacy and toxicity of systemic treatments: an undervalued issue in the era of precision oncology. J Clin Oncol.

[17] Anderson GD (2008). Gender differences in pharmacological response. Int Rev Neurobiol.

[18] Zucker I, Prendergast BJ (2020). Sex differences in pharmacokinetics predict adverse drug reactions in women. Biol Sex Differ.

[19] Moitra P, Chatterjee A, Kota PK (2022). Temozolomide-induced myelotoxicity and single nucleotide polymorphisms in the MGMT gene in patients with adult diffuse glioma: a single-institutional pharmacogenetic study. J Neurooncol.

